# Supplementary material for: Complexome profiling of the Chlamydomonas psb28 mutant reveals TEF5 as an early PSII assembly factor
Source: Plant Cell. 2025 Mar 17;37(6):koaf055. doi: 10.1093/plcell/koaf055 (PMC12164590; doi:10.1093/plcell/koaf055)
Supplement: koaf055_Supplementary_Data [file koaf055_supplementary_data.zip › psb28_interactive.zip!/plots/intensity/qualityPlots/peptides/_Cre48.g761197.t1.1.html]

### Cre48.g761197.t1.1

Trivial name:   
SeqCoverage [median]: 15.40  
Mass: 27.83 kDa  
  
 

| Sequence | MissedCleavage | Mass [Da] | wtIntensity | muIntensity | Score |
| --- | --- | --- | --- | --- | --- |
| EGEPVDIAGITVGK | 0 | 1383.72 | 0.00 | 2106.61 | 87.71 |
| TQDTDGYNAVQIGYK | 0 | 1671.77 | 0.00 | 359.07 | 155.02 |
| VGNVVEITPAK | 0 | 1125.64 | 964.03 | 1091.36 | 91.58 |
|  |  |  | 964.0 | 3557.0 |  |
